# Stereotactic body radiation therapy for liver tumours using flattening filter free beam: dosimetric and technical considerations

**DOI:** 10.1186/1748-717X-7-16

**Published:** 2012-02-01

**Authors:** Pietro Mancosu, Simona Castiglioni, Giacomo Reggiori, Maddalena Catalano, Filippo Alongi, Chiara Pellegrini, Stefano Arcangeli, Angelo Tozzi, Francesca Lobefalo, Antonella Fogliata, Piera Navarria, Luca Cozzi, Marta Scorsetti

**Affiliations:** 1 IRCCS Istituto Clinico Humanitas, Rozzano (Milano), Italy; 2 Oncology Institute of Southern Switzerland, Bellinzona, Switzerland

**Keywords:** SBRT, liver, flattening filter free, RapidArc, TrueBeam

## Abstract

**Purpose:**

To report the initial institute experience in terms of dosimetric and technical aspects in stereotactic body radiation therapy (SBRT) delivered using flattening filter free (FFF) beam in patients with liver lesions.

**Methods and Materials:**

From October 2010 to September 2011, 55 consecutive patients with 73 primary or metastatic hepatic lesions were treated with SBRT on TrueBeam using FFF beam and RapidArc technique. Clinical target volume (CTV) was defined on multi-phase CT scans, PET/CT, MRI, and 4D-CT. Dose prescription was 75 Gy in 3 fractions to planning target volume (PTV). Constraints for organs at risk were: 700 cc of liver free from the 15 Gy isodose, D_max _< 21 Gy for stomach and duodenum, D_max _< 30 Gy for heart, D_0.1 cc _< 18 Gy for spinal cord, V_15 Gy _< 35% for kidneys. The dose was downscaled in cases of not full achievement of dose constraints. Daily cone beam CT (CBCT) was performed.

**Results:**

Forty-three patients with a single lesion, nine with two lesions and three with three lesions were treated with this protocol. Target and organs at risk objectives were met for all patients. Mean delivery time was 2.8 ± 1.0 min. Pre-treatment plan verification resulted in a Gamma Agreement Index of 98.6 ± 0.8%. Mean on-line co-registration shift of the daily CBCT to the simulation CT were: -0.08, 0.05 and -0.02 cm with standard deviations of 0.33, 0.39 and 0.55 cm in, vertical, longitudinal and lateral directions respectively.

**Conclusions:**

SBRT for liver targets delivered by means of FFF resulted to be feasible with short beam on time.

## Introduction

Stereotactic body radiation therapy (SBRT) has proved its efficacy in several patient populations with primary and metastatic limited tumours [1]. In particular, SBRT may be appropriate for selected patients with oligo-metastatic disease, defined as less than five lesions [2] or with organ-confined limited volume primary tumours. Abdominal SBRT has been reported with reference mainly to primary and secondary liver tumours [1,3,4]. It is known that in the setting of limited tumour burden, SBRT leads to local control rates higher than 70%-80% [1], which may improve survival and quality of life.

RapidArc (RA) is a relative new VMAT technique based on simultaneous optimisation of multi leaf collimator (MLC) shapes, dose rate and gantry rotation speed [5]. The technology was investigated in several studies, showing comparable target coverage and a general improvement in organs at risk (OAR) and healthy tissue sparing, a reduced beam-on time and lower number of monitor units (MU) compared to other IMRT approaches [6-13]. In a previous work we demonstrated the feasibility and dosimetric advantage to use Volumetric Modulated Arc Therapy (VMAT) using RapidArc (RA) (Varian Medical Systems, Palo Alto, CA) in SBRT treatments of abdominal region to reduce treatment time, compared to 3D conformal radiotherapy (3D-CRT) and intensity modulated radiotherapy (IMRT) techniques [14]. Thus since November 2008 SBRT to abdominal targets has been delivered by means of RA. More recently, we reported our early experience in terms of technical feasibility, local control rate and acute toxicity profile of SBRT with RA for patients with primary or secondary abdominal tumours, showing the good toxicity profile and clinical results [15,16].

TrueBeam (Varian Medical System, Palo Alto, USA) is a new accelerator designed for delivering both in flattened filter (FF) and flattering filter free (FFF) modality [17]. In particular, the removal of flattening filter was shown to reduce of out-of-field dose due to the reduction of head scatter and residual electron contamination, consequence of the exclusion of beam attenuation due to flattened filter. Furthermore, with FFF beams the dose rate is increased up to a factor 4 for the 10 MV beam [18-23]. This leads to a possible reduction in delivery time with benefit in patient discomfort and with potential limitation of intra-fraction motion.

Some feasibility studies for SBRT using FFF beams are present in literature [23,24] and in a recent study performed in our institute we showed our early experience in the use of FFF beams for SBRT treatments including liver metastases, lung primitive and metastases, isolated abdominal lymph nodes, adrenal glands, and pancreas [25]. In the present study our attention was focused on the sub-group of patients that underwent a strongly hypo-fractionated treatment for which the time advantage is maximum. In particular our aim was to show a complete overview of SBRT liver protocol focusing on technical aspects: the deliverability is evaluated by gamma agreement index using two different devices (MatriXX and GafChromic), the analysis of the interfraction displacements by means of CBCT, and dosimetric objectives according to the number of lesions treated. The evaluation of radiation-induced liver disease (RILD), the radiobiological consequence due to the higher dose rate, and the clinical evaluation are not aim of this paper and these aspects will be evaluated in a specific study.

## Methods and materials

### Patients selection and treatment planning

Between November 2010 and September 2011 at Istituto Clinico Humanitas (ICH) 55 patients with 73 primary or metastatic liver tumours were treated by TrueBeam in an on-going phase II prospective protocol approved by the internal ethical committee. Aim of the protocol, approved in late 2009, was the evaluation of local control in patients with liver malignancies; secondary endpoints were the acute and late toxicities, and evaluation of overall survival. According with Fleming approach, to demonstrate local control of at least 80% with a power of 90%, at least forty-four patients in three years were necessary to complete the study. At present, all the patients are recruited and the follow-up are ongoing therefore no analysis regarding local control or toxicities will be reported in the present study.

Of the patients evaluated, 11 had primary liver tumours, and 44 had hepatic metastases from colon (23), biliar duct-pancreas (6), breast (5), and other sites (10). All the patients had been considered unfit for surgery or other non-surgical treatments at the time of radiation. Chemotherapy was stopped at least 3 weeks before SBRT and withheld until disease progression. Median patient age was 63 years (range: 43-83). Only patients with at least 1000 cm^3 ^of liver free from the disease and with ≤ than 3 lesions were considered eligible to the protocol.

Disease extension was evaluated in all cases by Computed Tomography (CT) with contrast. Although they were not inclusion criteria of the study, magnetic resonance imaging (MRI) and positron emission tomography/CT using FDG tracer (PET/CT) were acquired in respectively 2 and 14 patients. Both MRI and PET/CT were acquired for those patients whose treatment indication for radiotherapy was justified by these diagnostic exams; thus PET and MRI were only an integration in the target definition since the use of PET and MRI in defining the tumour is under evaluation and will be topic of future work. Computed tomography scans for planning were acquired for all patients positioned supine with their arms above the head; patients were immobilized by means of a thermoplastic body mask including a Styrofoam block for abdominal compression to minimize internal organ motion. Contrast free and 3 phases contrast-enhanced planning CT scans were acquired in free quiet breathing mode at 3 mm slice thickness with a stereotactic body frame to localise the isocentre. Breath hold in simulation CT was not mandatory in the study as many patients were unfit to maintain breath hold for many seconds with the compressor. In case of collaborative patients the simulations CT were performed in voluntary exhale breath hold.

Furthermore in case of lesions located in the VII or VIII hepatic segment or in case of liver cupola shift greater than 5 mm on the four simulation CTs, a four dimensional CT (4D-CT) was performed to best define the target margin. In addition, in two cases presenting internal clips due to previous surgery, the 4D-CT scan was acquired and the SBRT was performed in gated modality with internal marker tracking by 2D imaging. This option was released in July 2011 with TrueBeam version 1.5, and thus the first patients were not treated with internal marker tracking. This topic will be deepened in a specific paper.

The gross tumor volume (GTV) included macroscopic disease defined on CT as well as on PET if available. The clinical target volume (CTV) was defined equal to the GTV. The planning target volume (PTV) was generated by taking into account both the internal margin (IM) and the set-up margin (SM). Since SM was maintained at a minimum by the cone-beam CT (CBCT) daily verification of set up variations, the overall CTV-PTV margin was prescribed as 8-12 mm in the cranial-caudal axis and 4-6 mm in the anterior-posterior and lateral axes, allowing mainly for residual intra-fraction target motion as well as for inaccuracies in CBCT image interpretation [26,27]. The organs at risks (OAR) considered were: healthy liver, spinal cord, kidneys, stomach, duodenum, heart, small bowel, oesophagus and ribs, in relation with the lesion location.

The isodose distribution applied during SBRT typically includes planned heterogeneity within the tumor intended to intensify the dose within the tumor. In this protocol, the isodose line prescribed to cover the PTV was at least 67% of the prescribed dose (range 67-95%), trying to maximizing it up to 95% [28]. Dose prescription was set to 75 Gy in 3 consecutive daily fractions. For OARs, plans were required to meet the following objectives: V_15 Gy _(volume receiving 15 Gy) < (total liver volume - 700 cm^3^) for healthy liver, D_0.1 cm3 _for spinal cord < 18 Gy (dose at a volume of 0.1 cm^3 ^should be lower than 18 Gy), V_15 Gy _< 35% for both kidneys, V_21 Gy _< 1% for duodenum, small bowel, oesophagus, and stomach, V_30 Gy _< 1% for heart; D_30 cm_^3 ^< 30 Gy for ribs were considered as a secondary objective [3,29]. In case of overlap between PTV and duodenum or stomach, the priority was given to the OAR cropping the PTV to comply with the OAR limits.

### Treatment delivery

All plans were designed and optimised with RA technique using the optimizer PROIII for a Varian TrueBeam equipped with a Millennium multi-leaf collimator (MLC) with a leaf width of 5 mm at the isocentre. RA plans were designed using full (i.e. 360°) or partial (i.e. around 200°) multiple arcs according in order to achieve the best dose distributions. Specifically, partial arcs were used in cases of (1) lesion located far from the median axis (i.e. more than 10 cm) to do not collide the gantry with the couch induced by laterality of the couch, and (2) lesion very close to serial OARs (i.e. heart, gastro-intestinal organs) to best protect them. Where possible, coplanar arcs were employed to fasten the delivery time, otherwise, non coplanar arcs arrangements were used with two perpendicular couch positions. In particular, the non-coplanar approach was adopted in multi-lesions cases only. All dose distributions were computed with the Analytical Anisotropic Algorithm (AAA) (version 10.0.28) implemented in the Eclipse planning system with a calculation grid resolution of at maximum 2.0 mm.

Treatment was delivered in 3 consecutive working days, with the patient keeping a 3-hour fast to avoid gross displacement of stomach. Treatment delivery included stereotactic frame localization in the first session aiming to a preliminary isocentre positioning followed by image guidance with on-line couch adjustment at each fraction by means of cone beam CT (CBCT). Couch repositioning was operated after automatic matching of CBCT images to reference planning CT, followed by manual refining. The shift values were analyzed for all patients and random (σ) and systematic (Σ) population errors were calculated according to Van Herk approach [30]. In two cases the delivery was performed in respiratory gated modality. This approach allows the radiation beam to be turned off when respiratory movements place the target outside of the predetermined positioning parameters, and to resume the radiation when the target falls back within the accepted alignment. In particular, the respiration path was revealed with RPM system (Varian) and internal markers, previously detected on the simulation CT. The markers were detected by instantaneous kV-portal images acquired before each beam-on phase. On each of these kV-portal images, a circular region of interest (ROI) of 5 mm radius defined the theoretical marker's position for each projection; the radiation oncologist could then verify the instantaneous marker's positions to be inside the ROI, highlighting possible internal organ motions.

Before treatment, each plan was verified to assess dosimetric agreement between computed and delivered dose distributions. This quality assurance process was performed with two independent quality assurance systems (MatriXX and Gafchromic). The results of these measurements were scored in terms of the Gamma Agreement Index (GAI) based on the γ of Low analysis [31] with thresholds: Distance to Agreement = 3 mm, ΔDose = 3%.

### Data analysis and statistics

Technical parameters of delivery were scored in terms of number of arcs, total number of monitor units (MU), monitor units per Gy (MU/Gy), total beam on time, total treatment time (the time in which the patient is in the bunker), and isocenter shift. Dosimetric quality of treatments was measured on the basis of dose volume histogram (DVH) analysis. For CTV and PTV the following data were reported: target coverage (mean, D_1%_, D_95%_, V_95%_, V_107%_) and conformity for PTV. Conformity index (CI_95%_) was defined as the ratio between the volume of patient irradiated at 95% of the prescribed dose and the target volume. For OARs, the mean dose, the maximum dose (D_xcm3_) and appropriate values of V_xGy _(volume receiving at least x Gy) were scored. The data were reported separately for patients with 1 lesion and with 2-3 lesions. The Kolmogorov-Smirnoff test was applied to the data to evaluate if the data were normally distributed.

## Results

**Figure 1:**
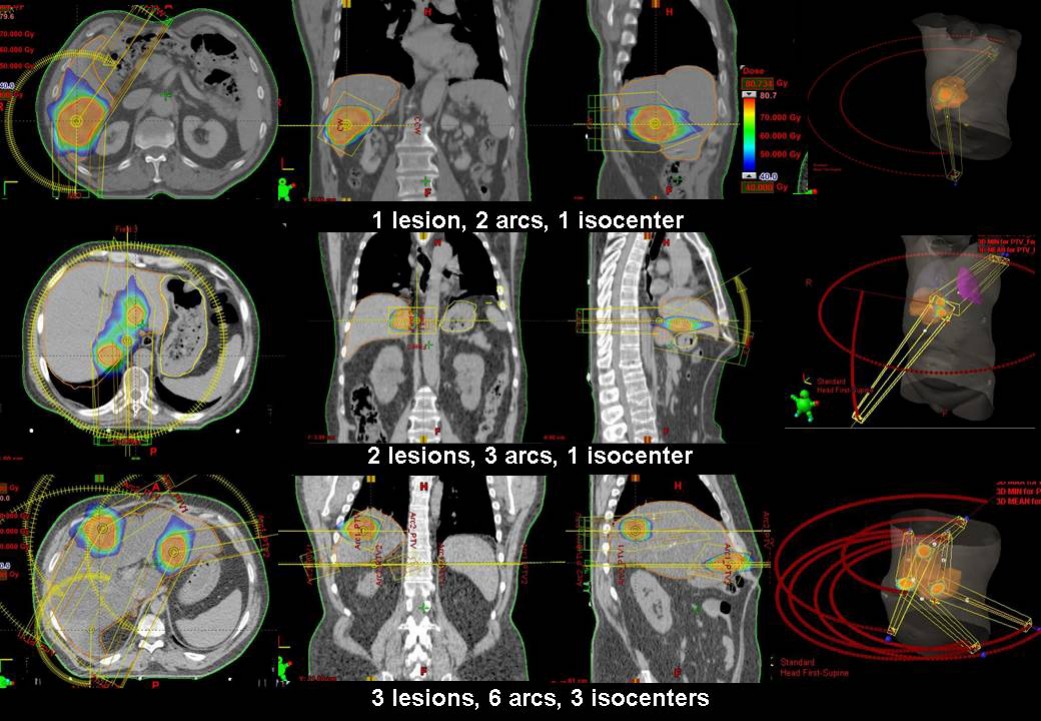
**Examples of dose distributions**. Colourwash scale is reported on the figure. Solid lines represent target volumes and organs at risk.

Forty-three patients with a single lesion, nine with two lesions and three with three lesions were treated in this protocol, for a total of 73 isocenters. Figure 1 shows examples of dose distributions for three representative patients. Similar results were obtained in all patients. PTV and organs at risk are outlined as solid lines in the images. Table 1 reports the summary of dosimetric results.

**Table 1:** summary of dosimetric results for CTV, PTV and organs at risk

	CTV		PTV		HT		Spine		Ipsilateral Kidney		Liver	
	1 les	2-3 les	1 les	2-3 les	1 les	2-3 les	1 les	2-3 les	1 les	2-3 les	1 les	2-3 les
**Vol (cm^3^)**	22.3 ± 14.3	18.6 ± 15.4	71.2 ± 33.6	72.2 ± 45.8	-	-	43.7 ± 26.7	46.0 ± 19.0	156.1 ± 40.6	154.0 ± 53.4	1349.1 ± 238.4	1708.0 ± 940.7
**Mean (Gy)**	75.9 ± 1.1	75.8 ± 0.6	72.9 ± 4.1	71.5 ± 4.4	30.0 ± 5.7	30.4 ± 2.6			3.4 ± 4.2	4.1 ± 3.8	15.1 ± 4.4	16.0 ± 6.9
**D_0.1 cc _(Gy)**	-	-	-	-	-	-	9.8 ± 4.6	12.3 ± 4.2	-	-	-	-
**D_1% _(Gy)**	77.8 ± 1.3	77.9 ± 1.0	77.4 ± 1.6	77.4 ± 1.1	-	-	-	-	-	-	-	-
**D_95% _(Gy)**	73.7 ± 1.4	74.0 ± 0.8	66.7 ± 9.6	64.7 ± 9.8	-	-	-	-	-	-	-	-
**V_10 Gy _(%)**	-	-	-	-	87.2 ± 17.1	85.3 ± 3.6	-	-	-	-	-	-
**V_15 Gy _(cm^3^)**	-	-	-	-	-	-	-	-	10.0 ± 15.7	7.2 ± 8.9	384.2 ± 194.2	425.3 ± 234.2
**V_67% _(%)**	-	-	99.3 ± 2.3	99.8 ± 0.3	-	-	-	-	-	-	-	-
**V_80% _(%)**	-	-	92.8 ± 11.9	89.0 ± 14.2	-	-	-	-	-	-	-	-
**V_95% _(%)**	99.4 ± 1.56	99.5 ± 0.45	81.6 ± 26.9	73.0 ± 32.4	-	-	-	-	-	-	-	-
**V_107% _(%)**	0.0 ± 0.0	0.0 ± 0.0	0.1 ± 0.2	0.0 ± 0.0	-	-	-	-	-	-	-	-
**CI_95%_**	-	-	1.1 ± 0.2	1.3 ± 0.5	-	-	-	-	-	-	-	-

The Kolmogorov-Smirnoff test revealed the data were normal distributed, thus the mean and standard deviation were used to present the results. In seven patients (i.e. 12% of cases) a 20% dose downscaling was necessary to comply with the liver constraint (V_15 Gy free _> 700 cm^3^). In all cases the maximum dose resulted satisfactory with negligible PTV volumes exceeding 107%. Planning objectives for organs at risk were largely met in most of the cases for both groups of patients. The always resulted > 700 cc as requested by the constraints, with mean and SD values of 965 ± 141 cc and 1283 ± 706 cc for single and multi lesion patients respectively. The higher V_15 Gy free _liver value for multi lesion patients was due to the larger liver volumes of this cohort of patients; the high mean and SD values are ascribable to a single case in which the total liver volume was 3320 cc (excluding the case, the mean value decreases to 1039 ± 157 cc).

**Figure 2:**
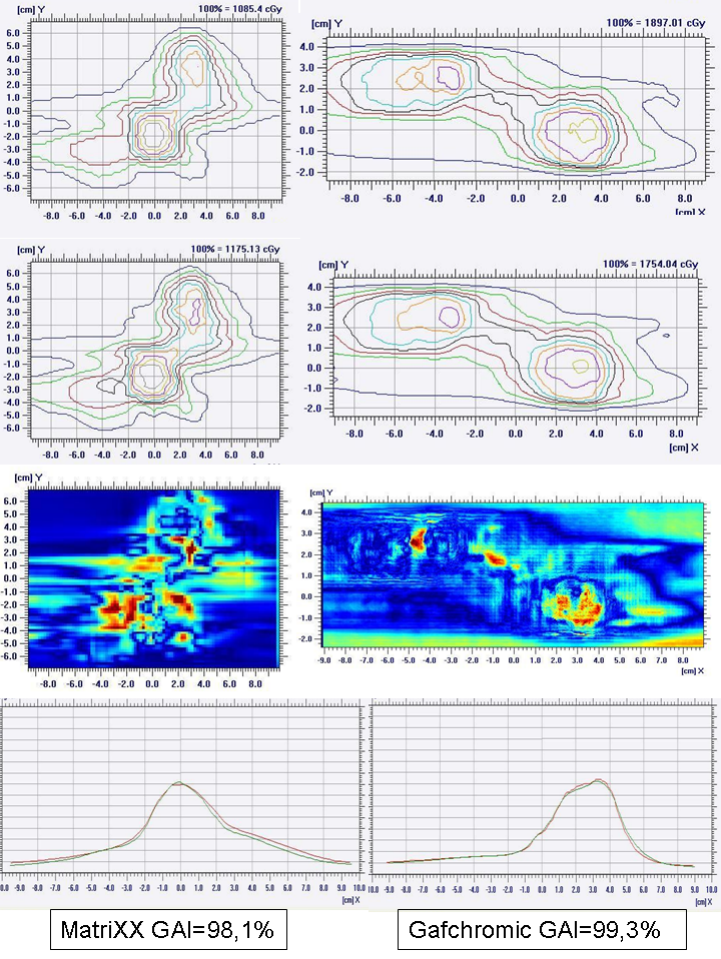
**GAI evaluation for a 2 lesion case using (a) Gafchromic and (b) MatriXX approaches**.

Pre-treatment quality assurance measurements resulted satisfactory with a minimum deviation between groups of less than 3% and no statistically significant differences (98.7 ± 1.1% and 97.9 ± 1.1% for single and multi lesion patients respectively). In all cases GAI exceeded the acceptance threshold of 95%. Figure 2 shows an example of GAI analysis for a 2 lesions case using both Gafchromic and MatriXX.

Patients with 1 lesion were all treated with 2 full or partial arcs while patients with 2-3 lesions were treated with 2 to 6 arcs (2.7 ± 1.5). The total MUs employed were 5241 ± 1238 for single lesion plans and 7550 ± 3594 for the others, resulting in beam on time respectively of 2.3 ± 0.5 min and 3.2 ± 1.5 min, with global range of 1.9-6.2 min (this last case was a three lesion with six arcs plan). The overall treatment time, including positioning, imaging, repositioning, and delivery time, was 16.2 ± 1.7 min for the first fraction, lowering to 12.4 ± 1.5 min for the subsequent ones. Average MU/Gy resulted in the order of 200-250, slightly increasing for multi-lesion patients (≈300), confirming the intrinsic efficiency of RA technique also in terms of machine output factor.

**Figure 3:**
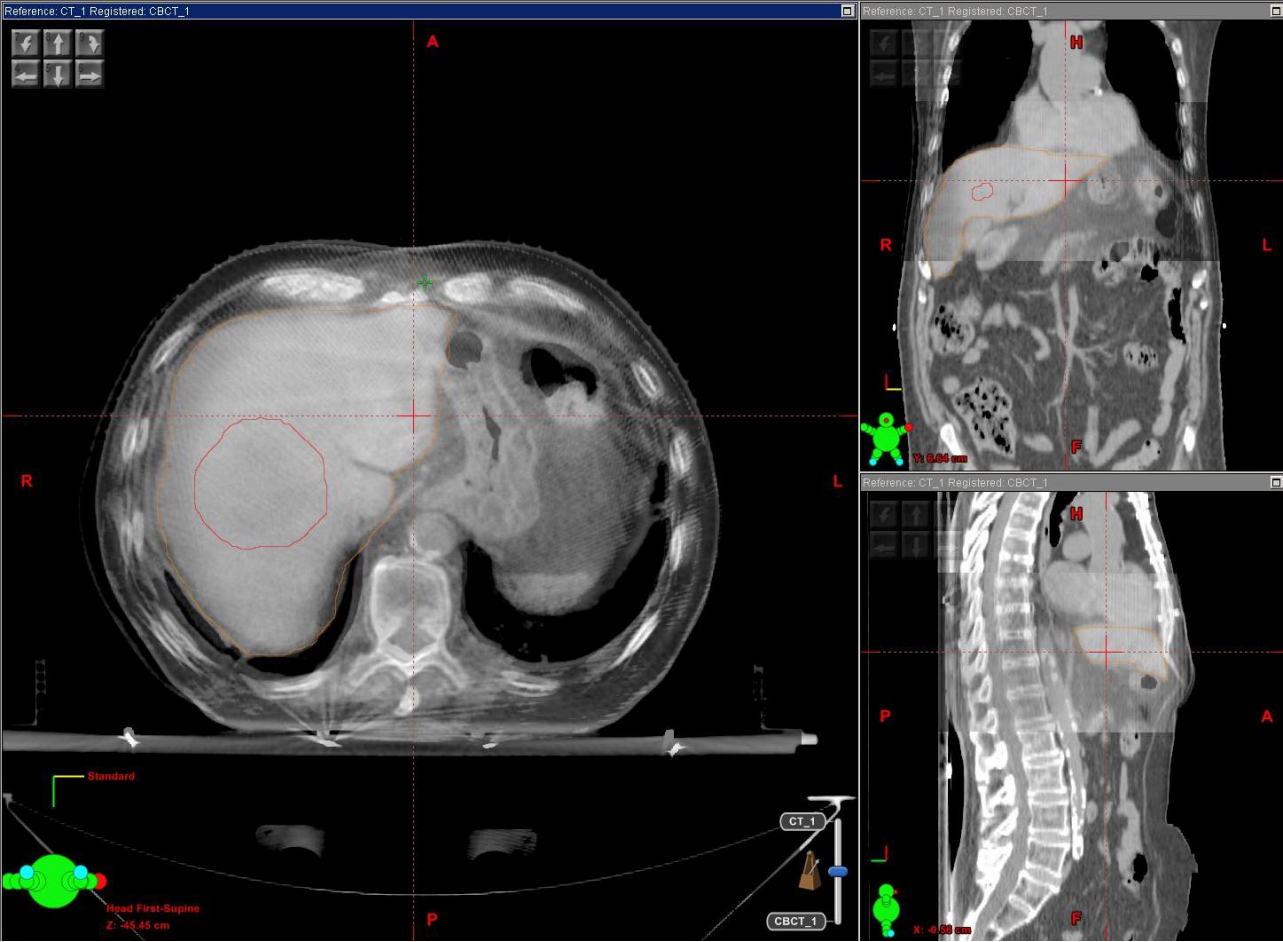
**Daily CBCT matching with the simulation CT for the repositioning**.

Figure 3 shows a CBCT matching with the simulation CT for a patient with a single liver lesion. An analysis of the corrections applied by means of a daily CBCT was performed including all the 219 CBCT acquired for these patients, in order to assess any systematic error due to an inaccurate patient positioning. The mean displacements found were -0.08, 0.05 and -0.02 cm with standard deviations (SD) of 0.33, 0.39 and 0.55 cm in vertical, longitudinal and lateral directions respectively. The random (σ) and systematic (Σ) population errors, as defined in [30] were calculated with the following results: σ = 0.25-0.26-0.34 cm, Σ = 0.20-0.25-0.46 cm.

## Discussion and Conclusions

The use of SBRT for the irradiation of primary and metastatic tumours in several anatomical sites is becoming a standard of treatment. SBRT has emerged as a possible non invasive approach for local ablation in some series of selected cancer patients. In particular, in abdominal sites SBRT for patients with either organ-confined primary tumours or oligo-metastatic disease may play a major role for improving survival in a clinically significant subset of cancer patients. Although in most cases the radiation sterilization of a metastatic lesion is not expected to lead to definitive cure, it could be effective in delaying further chemotherapy or at least this may contribute to better quality of life and local control.

The high dose per fraction of SBRT induces a huge amount of MU is required to cover the target and sparing the neighbour organs at risk, with a consequent increase of the beam on time with respect to the standard RT of 2 Gy/day. In this context, the recent introduction of volumetric modulated arc therapy, e.g. RapidArc, has shown to reduce the total monitor units and thus the treatment time in comparison with intensity modulated RT (IMRT) without compromising the target coverage as shown in many anatomical regions.

In this paper we considered the first 55 consecutive patients that underwent SBRT on both primary and metastatic hepatic targets, using FFF beams. In particular 43 patients presented a single lesion, 9 two lesions and 3 presented three lesions. Removal of the flattening filter gives the possibility to deliver treatments with higher dose rates, up to a factor 4 at 10 MV, and with a much higher dose per pulse. The different physical characteristics of the FFF beams (e.g. the different beam profile) are under investigation by the scientific community [18-23] and first preliminary clinical data are in progress [25].

In a previous report we analyzed a cohort of patients treated with RapidArc technique for abdominal lesions, including abdominal lymph-nodes, pancreas, and liver metastases [16]. All plans were optimized using flattened beams. In particular, comparing the results obtained for the liver metastases cohort in [16] and the data reported in this study it appears that, though for all patients 95% of the PTV volume received at least 67% of the prescription dose, more patients could receive higher mean doses to the PTV with FFF beams: mean dose to PTV was 67 Gy in [16] and around 73 Gy in the present study, PTV V95% passed from 40% to 81%, of course the higher doses to the target implies higher doses to OARs (e.g. liver V15Gy passed from 257 to 384 cc) but the constraints were always respected, the mean GAI value improved passing from 97.8% to 98.7%. On this topic, in a recent paper, we specifically compared FFF and FF beams from a dosimetric prospective, demonstrating by a theorical point of view FFF beam to be adequate in abdominal SBRT for lesions from small to medium sizes (i.e. up to 200-300 cc), with adeguate healthy tissue sparing and PTV coverage with respect to flattened beams [32]. The only significant variation between the two cohorts was the beam on time. In the previous study, where only single hepatic metastases were considered, around 9 minutes were necessary to deliver the treatment while in the present group of patients the beam on time was reduced to around 2.3 minutes (considering only the single-lesion patients), with a cut of time of more than 350%, decreasing patient's discomfort and reducing intra-fraction uncertainties. In both series the instantaneous delivery dose rate was almost always at the maximum value, 600 MU/min for FF beams and 2400 MU/min for FFF, hence the beam on time reduction. Furthermore the data reported in the present study showed that plans for with multi-lesion patients are dosimetrically comparable with the single lesion ones.

In our Institution, image-guidance by means of CBCT, implemented in the therapeutic radiation device, is daily used to better define and correct setup of each patient before each fraction of the treatment. The analysis of the daily displacements, in particular the mean values very close to 0, show the efficiency of our set-up method; however, the calculation of Σ and σ and of the relative margins, shows the absolute necessity of daily repositioning by means of CBCT; the efficiency of the set-up alone with abdominal compression and stereotactic body frame is not sufficient to apply margins of 5-8 mm in the anterior-posterior and lateral axes. Daily Image guidance has therefore allowed the minimizing of the set-up margins from CTV to PTV, reducing the normal tissue surrounding the target close to healthy organs in critical sites, such as in abdomen. At this purpose, Eccles et al. from Princess Margaret Hospital showed, in case of daily CBCT linked with abdominal compression, the maximum changes in tumour center of mass to be lower than 5 mm in 94% patients, with mean displacement of 1.4 mm, 2.1, and 1.0 mm in, respectively, LR, AP, CC directions [25].

In conclusion, we reported our practice in the treatment of liver metastases on the first 55 patients using FFF beams, prescribing 75 Gy in 3 fractions at PTV. In the majority of cases the beam on time was lower than 3 minutes, strongly reducing the treatment time in comparison with flattened filter beams, without compromising the target coverage and organs at risk sparing.

## Competing interests

Dr. L. Cozzi is Head of Research at Oncology Institute of Southern Switzerland and acts as a Scientific Advisor to Varian Medical Systems. Other authors have no conflict of interest.

## Authors' contributions

GR, MC, CP, SA, FL, AT, AF and LC carried out the data and participated in the data evaluation. PM, SC and GR drafted the manuscript. PM, SC and GR participated in the design of the study. FA, AF, PN and LC performed the statistical analysis. SC, FA, SA, AT, PN and MS carried out the patients record evaluation and followed patients and treatments. The definitive supervision of the paper was done by PM and MS. All authors read and approved the final manuscript.
